# Phylogenetic Relationships and Disturbance Explain the Resistance of Different Habitats to Plant Invasions

**DOI:** 10.3390/life12111785

**Published:** 2022-11-04

**Authors:** Chaodan Guo, Caiyun Zhao, Feifei Li, Jianfeng Huang

**Affiliations:** 1 Institute of Ecology, Chinese Research Academy of Environmental Sciences, Beijing 100012, China; 2 The Administration Bureau of Encheng National Nature Reserve, Chongzuo 532200, China

**Keywords:** invasive alien plants, protected areas, habitat invasibility, interference hypothesis, Darwin’s naturalization hypothesis

## Abstract

Invasive alien plants have invaded various habitats, posing a threat to biodiversity. Several hypotheses have been proposed to explain the mechanisms of invasion, but few studies have considered the characteristics of the invaded communities and the effects of human interference in the invasion. In this study, we compared the invasibility of three different habitats: abandoned land, eucalyptus plantations, and natural secondary forests. We explored the effects of species diversity, phylogenetic diversity, and disturbance factors on the invasibility of different habitats. The results showed that the invasibility of abandoned land was the highest and the invasibility of the natural secondary forest was the lowest. Phylogenetic indicators affected the invasibility of abandoned land and eucalyptus plantations, and disturbance factors affected the invasibility of all three habitats, while the characteristics of the invaded communities had a weak impact. Our research provided supporting evidence for Darwin’s naturalization hypothesis and his disturbance hypothesis but found no relationship between biotic resistance and invasibility. This study indicated that the differences among habitats should be considered when we prove Darwin’s naturalization hypothesis in nature reserves.

## 1. Introduction

As trade and transport have increased in intensity over time, so too has the number of invasive alien species (IAS) [1]. IAS are one of the most serious threats to biodiversity, causing changes in species, community structure, and ecosystem function [2,3]. Understanding the mechanisms of the successful invasion of alien species is a key step in predicting and preventing the invasion of potentially invasive alien species [4,5]. Invasibility of habitats and the invasiveness of alien species are key factors in understanding successful invasion and are also hot topics in invasion ecology [6,7].

Several hypotheses have been proposed to explain the biotic resistance of a recipient plant community, including the classical biotic resistance hypothesis, the interference hypothesis, and Darwin’s naturalization hypothesis [8]. The biotic resistance hypothesis is supported by the fact that ecosystems with higher biodiversity are more resistant to invaders than those with low biodiversity [9,10]. A review study found that only 29% of studies supported the theory, and support for this theory has declined as more studies have been published [11]. Other researchers have attributed differences in findings to spatial scales and disturbances [12,13,14]. The disturbance hypothesis emphasizes that environmental changes caused by natural or human disturbance produce ecological niches suitable for the survival of alien species, thus promoting invasion [15].

Darwin [16] suggested that alien species with closer phylogenetic relationships with native species would be more likely to unsuccessfully invade compared to more unrelated alien species, as they have a higher degree of overlap in niches, and are therefore more competitive [17]. Darwin [16] also recognized that alien species closely related to native species were more likely to be naturalized because they had the same pre-adaptation to local environmental conditions as the native species [18]. In light of this argument, increasing the phylogenetic relatedness between an alien species and its recipient community will increase its probability of invasion success [19]; this is known as the pre-adaptation hypothesis. The antithesis of the pre-adaptive hypothesis and Darwin’s naturalization hypothesis is now known as “Darwin’s naturalization conundrum” [20]. Some ecologists’ studies have partly supported the pre-adaptation hypothesis, some studies have supported Darwin’s naturalization hypothesis, and some studies have not reached clear conclusions [21,22,23,24]. Although ecologists have tried to explain the divergence between different studies in terms of spatial scale [25] and invasion stage [26], there are still great difficulties in reconciling this mystery. Few studies have considered, at the same time, biotic resistance, disturbance, and phylogenetic diversity in a community. 

Nature reserves are considered to be the cornerstone of biodiversity conservation, but under the human pressure of the current Anthropocene era, the risk of biological invasion in nature reserves is also increasing [27,28]. Many invasive plants have been observed in nature reserves in Central Europe [29], Africa [30], and across the world [31,32]. Although the increase of biodiversity and ecosystem function in nature reserves is expected to prevent biological invasions according to “the biotic resistance hypothesis” [9,10], recent research has reported that native plant species richness and human population density are positively related to the presence of alien plants species in nature reserves in Greece [33]. More evidence from different nature reserves is still needed. Nature reserves are rich in biodiversity and provide an ideal research site for demonstrating the hypothesis of biological invasion. Here, we selected three habitat types in the Encheng National Nature Reserve: abandoned land, eucalyptus plantations, and natural secondary forest, which represented different native species diversity and human disturbance. We examined the impacts of taxonomic species diversity, human disturbance, and phylogenetic diversity on the invasibility of different habitats within the nature reserve. We aimed to determine: (a) whether there were differences in the invasibility of different habitats in the nature reserves and (b) the main impact factors of the invasibility of different habitats.

## 2. Materials and Methods

### 2.1. Study Area

The Encheng National Nature Reserve (106°58′16″–107°15′36″ E, 22°36′29″–22°50′05″ N) is located in southwestern Guangxi Zhuang Autonomous Region, China, with an area of 25,819.6 hm^2^. The regional climate is subtropical monsoon type, with a mean temperature of 22.3 °C and mean annual precipitation of 1362 mm, which is mainly concentrated from May to September [34]. The main landforms in the reserve are karst peak clusters, peak forests, and isolated peaks, accounting for about 85% of the area of the reserve. The population density in the area is 39 persons/km^2^. Arable land in the reserve covers 18.62% of the total area and is mainly planted with crops such as *Saccharum officinarum* L., *Zea mays* L., *Musa nana* Lour., *Hylocereus undatus* (Haw.) Britt. et Rose, and *Citrus reticulata* Blanco. The area of forest land accounts for 80.3% of the total area of the reserve, of which 1.06% is planted with eucalyptus forest. Protection of *Trachypithecus francoisi* Pousargues, 1898 and other rare and endangered wildlife and their habitats, as well as the northern tropical karst forest ecosystem, are the main targets of Encheng National Nature Reserve.

### 2.2. Study Design and Species Survey

Field data were collected in September 2020, and three habitat types near the road were randomly selected for the vegetation survey: AL (abandoned land), EP (eucalyptus plantation) and NF (natural secondary forest). See Table 1 for details of sample points.

**Figure 1:**
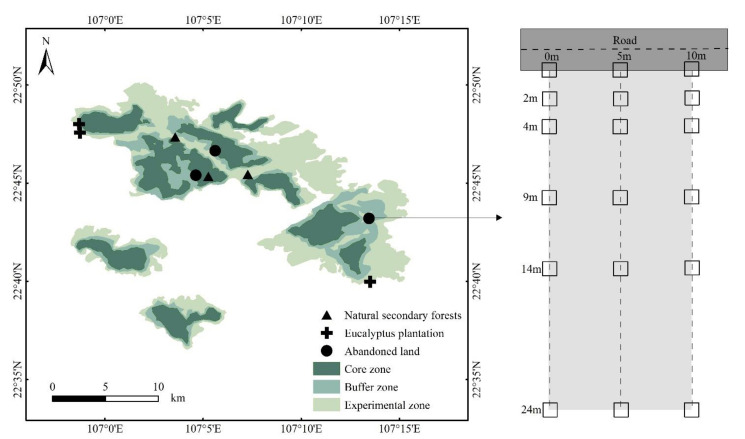
Sample design in this study.

A sample plot of 10 m × 24 m was set up for each habitat (Figure 1). In each sample plot, one 10 m long sample strip was set parallel to the road; then three parallel strips were set perpendicular to the parallel strips at the beginning, middle, and end, and one 1 m × 1 m subplot was set at different distances from the road shoulder: 0 m, 2 m, 4 m, 9 m, 14 m, and 24 m on each strip. A total of 18 subplots was conducted in each plot. Plant species (including herbs, trees, and shrub seedlings) and plant numbers, plant heights, and plant cover were recorded in the subplots. 

All plant species were identified by botanical experts. The plants recorded were classified as invasive alien plants and native plants based on expert identification and the “The Checklist of the Alien Invasive Plants in China” [35]. 

We also collected the road grade, road width, and road type of each habitat during the vegetation survey. The nearest village distance and village population were provided by the reserve staff. During the vegetation survey, we also recorded the traffic and human flow during one hour in each habitat and then we calculated the average traffic and human flow (the total number/time).

### 2.3. Response Variable and Explanatory Variables

#### 2.3.1. Response Variable

The RA (relative abundance) of invasive alien plants was used as an indicator of habitat invasibility. RA was calculated using the following equation [36]:

$$\mathrm{RA}=\frac{\mathrm{Number}\;\mathrm{of}\;\mathrm{individuals}\;\mathrm{of}\;\mathrm{invasive}\;\mathrm{alien}\;\mathrm{plants}}{\mathrm{Density}\;\mathrm{for}\;\mathrm{all}\;\mathrm{species}}\;\times \;100\%$$

Larger RA values indicated higher invasibility of the habitat. We organized data in R using the acast function from package “reshape2” and calculate RA in Microsoft Excel 2019 (https://www.microsoft.com/zh-cn/microsoft-365/excel).

#### 2.3.2. Explanatory Variables

There were two types of explanatory variables in our study: (1) biotic factors: species richness of native plants; phylogenetic diversity including MPD (mean pairwise phylogenetic distance) and MNTD (mean nearest taxon distance) between invasive alien plants and native plants; and SES.MPD (standardized effect sizes of mean pairwise distance) and SES.MNTD (standardized effect sizes of mean nearest taxon distance) of native plants [37] and (2) disturbance variables: average traffic flow, average human flow, road grade, road type, road width, village distance, and village population. 

To measure the phylogenetic relationships, conserved chloroplast regions (matK) and the nuclear ribosomal ITS region for all available species were retrieved from NCBI (https://www.ncbi.nlm.nih.gov/, accessed on 21 June 2021) to reconstruct the phylogeny. Fern species were excluded due to the large phylogenetic distance between ferns and angiosperms, which would have affected the precision of the analysis [38]. Our final sample sizes were 11 invasive alien plants and 94 native plants. Sequences of each region were aligned independently with the MAFFT version.6 (https://mafft.cbrc.jp/alignment/software/, accessed on 5 July 2021) [39,40], cut manually using BioEdit [41], and combined into a single alignment matrix with MEGAX [42]. Finally, we used MEGAX to construct the phylogenetic tree by the neighbor-joining method, using the Kimura2-parameter model. Node support was assessed by bootstrap analysis with 1000 replicates.

We measured SES.MPD and SES.MNTD [37] of native plants as:

SES.MPD = (MPD_obs_ − meanMPD_rand_)/sdMPD_rand_

SES.MNTD = (MNTD_obs_ − meanMNTD_rand_)/sdMNTD_rand_

where MPD_obs_ or MNTD_obs_ are the observed MPD or MNTD, meanMPD_rand_ and meanMNTD_rand_ are the mean of the MPD or MNTD values, respectively, obtained for the null species pools; and sdMPD_rand_ and sdMNTD_rand_ are the standard deviation of the MPD or MNTD values, respectively, of the null species pools [43]. Since the phylogenetic diversity index is correlated with species richness to some extent [44], we assessed using standardized effect sizes obtained by the null models. We randomized the communities by shuffling the tip labels of the phylogeny 999 times; this randomization maintained the species richness and occupancy [45]. For these and all subsequent analyses, we removed all plots with a single species. Both indices were calculated in R using functions SES.MPD and SES.MNTD from package picante [46].

MPD is generally thought to be more sensitive to tree-wide patterns of phylogenetic clustering and evenness, while MNTD is more sensitive to patterns of evenness and clustering closer to the tips of the phylogeny [47]. Using both indices enabled us to assess the effect of phylogenetic relatedness on two phylogenetic scales. We measured MPD and MNTD as:

$$\mathrm{MPD}=\frac{\sum_{i=1}^{n_{k_{1}}}{\;\bar{\delta }}_{ik_{2}}+\sum_{j=1}^{n_{k_{2}}}{\;\bar{\delta }}_{jk_{1}}}{n_{k_{1}}+n_{k_{2}}}$$

$$\mathrm{MNTD}=\frac{\sum_{i=1}^{S_{A}}\min\delta_{\mathrm{iB}}+\sum_{j=1}^{S_{B}}\min\delta_{\mathrm{jA}}}{S_{A}+S_{B}}$$

where ${\;\bar{\delta }}_{ik_{2}}$ is the mean pairwise phylogenetic distance between species i in community $k_{1}$ and all species in community $k_{2}$, $n_{k_{1}}$ represents the number of species in community $\;k_{1}$, $S_{A}$ is the number of species in community A, and $\min\delta_{\mathrm{jA}}$ represents the phylogenetic distance between species j in community B and its closest relative species in community A [37]. First, we divided each plot into two, one containing only invasive alien plants and the other containing only native plants. Finally, the MPD and MNTD between invasive alien plants and native plants were filtered from the results. Both indices were calculated in R using functions comdist and comdistnt from package picante [46].

To investigate differences between the IAP (invasive alien plants) and NP (native plants) in diversity across the three study habitats, we calculated species richness, Shannon–Weiner diversity, Simpson dominance, and the Pielou evenness index. Species diversity indices were calculated using the R package “vegan” [48].

### 2.4. Data Analyses

The diversity of native plants and invasive alien plants among three habitats was assessed using one-way ANOVA. We used backward and forward stepwise variable selection to find a model of habitat invasibility and explanatory variables [49]. The Akaike information criterion was used as the model performance metric [50]. In the process of stepwise regression, we introduced new variables one by one, and considered whether to eliminate the selected variables when introducing a new variable until it is no longer necessary to introduce new variables. R^2^ and MSE (mean squared error) were used to evaluate the accuracy of the regression model [51]. A value of R^2^ closer to 1 indicates that the regression model fits the observation. The MSE is the mean of the sum of the squares of the residuals, and a smaller MSE indicates that the resulting model error is smaller [52]. MASS package in R was used to perform stepwise regression analysis.

## 3. Results

### 3.1. Species in Different Habitats 

A total of 12 invasive alien plants were recorded, belonging to 8 families and 12 genera. The highest richness of invasive alien plants was found in EP. Asteraceae had the largest number of invasive alien plants, with five species, accounting for 41.67% of all species; there was one species from each of the other families. The invasive alien plants were mainly annual herbs, with a few perennial herbs and shrubs or subshrub-like herbs, and the majority of invasive alien plants originated from America (Table 2). *Chromolaena odorata* (L.) R. M. King & H. Robinson and *Bidens pilosa* L. were common species in all three habitats. A total of 135 native plants was recorded, belonging to 52 families and 110 genera, of which 47 species were recorded in in AL, 47 species in Ep, and 67 species in NF.

### 3.2. Species Diversity in Different Habitats

**Figure 2:**
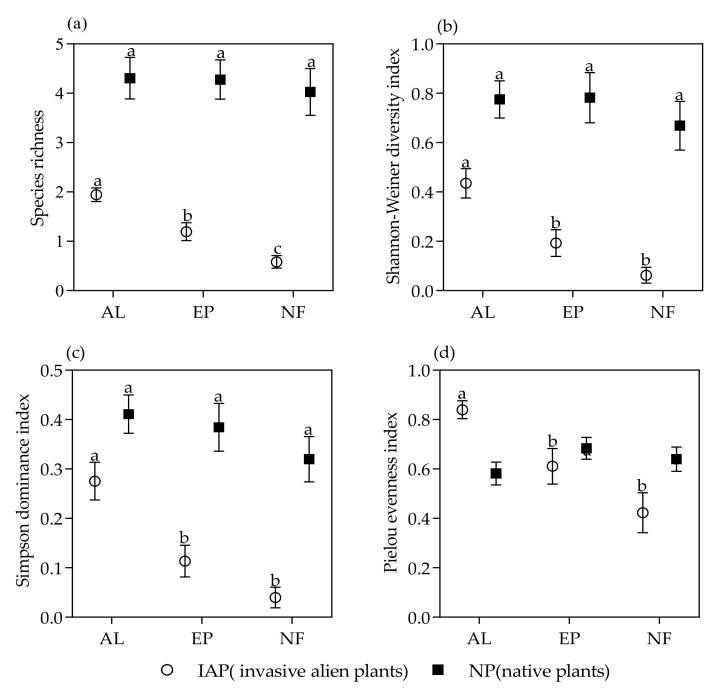
Mean ± SE of the diversity index of NP and IAP in different habitats. (**a**) species richness of NP and IAP; (**b**) Shannon–Weiner diversity of NP and IAP; (**c**) Simpson dominance of NP and IAP, (**d**) Pielou evenness of NP and IAP. Different lowercase letters indicate significant differences. IAP and NP represented invasive alien plants and native plants, respectively. AL, EP, and NF represented abandoned land, eucalyptus plantations, and natural secondary forests respectively.

One-way ANOVA results showed significant differences in species richness (F = 20.362, *p* < 0.001), Pielou’s index (F = 10.044, *p* < 0.001), Shannon’s index (F = 14.35, *p* < 0.001), and Simpson’s index (F = 14.978, *p* < 0.001) in different habitats. Multiple comparisons showed that the species richness of invasive alien plants in AL was markedly higher than that in EP, while that in the NF was significantly lower than that in EP (Figure 2a). The Shannon–Weiner diversity (*p* < 0.01) (Figure 2b), Simpson index (*p* < 0.01) (Figure 2c), and Pielou evenness (*p* < 0.01) (Figure 2d) of invasive alien plants in AL were significantly higher than those in the other two habitats, and these three indexes of invasive alien plants in EP were slightly higher than those in NF but no significant difference was found. No statistically significant (*p* > 0.05) differences in the diversity index of native plants among habitats were observed. The diversity index of native plants in the three habitats was higher than that of invasive alien plants.

### 3.3. Invasibility in Different Habitats

**Figure 3:**
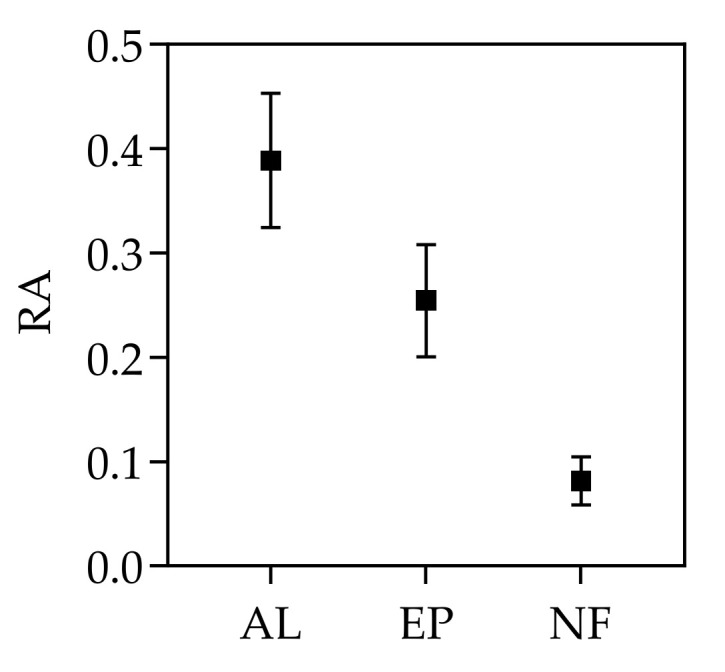
Comparison of invasibility in different habitats. RA represented the relative abundance of alien invasive plants in each habitat. AL, EP and NF represented abandoned land, eucalyptus plantation and natural secondary forest, respectively.

The highest invasibility was found in abandoned lands and the lowest was in natural secondary forests. The RA of invasive alien plants in AL was 0.389 ± 0.064, RA of invasive alien plants in EP was 0.254 ± 0.054, and the RA of invasive alien plants in NF was 0.082 ± 0.023 (Figure 3).

### 3.4. Model Results of Habitat Invasibility

A total of 12 explanatory factors belonging to three types and habitat invasibility were analyzed by stepwise linear regression, and the R^2^ and MSE values of the model were obtained. The results showed that the R^2^ of the stepwise linear regression model of the AL, EP and NF was 0.852, 0.588 and 0.339, respectively. The MSE values were 0.019, 0.038, and 0.011, respectively. 

The results of stepwise regression analysis showed that the influencing factors affecting the invasibility of different habitats were different (Table 3). In AL, habitat invasibility was significantly negatively correlated with SES.MPD, average traffic flow and average human flow, but positively correlated with SES.MNTD. In EP, habitat invasibility was significantly positively correlated with MNTD and average traffic flow but was negatively correlated with the average human flow. In NF, habitat invasibility was positively correlated with average traffic flow but negatively correlated with the average human flow. From the results, disturbance variables were found to have a significant effect on the invasibility of all three habitats, while phylogenetic indicators had a significant effect on AL and EP, and native plants species diversity of invaded habitats had no effect on the invasibility of all three habitats.

## 4. Discussion

Our study found that invasibility was highest in AL but lowest in the NF. Disturbance factors constantly impacted the invasibility of the three habitats. Phylogenetic factors affected the invasibility of AL and EP. Native plant diversity had less influence on the invasibility of the three habitats. Our results supported the disturbance hypothesis and Darwin’s naturalization hypothesis, and no supporting evidence for the biological resistance hypothesis was found. 

SES.MPD was negatively related to the invasibility of AL but SES.MNTD was positively related to it. Our results indicated that the more cluster in the phylogenetic structure of native communities in AL, the higher the invasibility of habitat. This is consistent with previous studies that showed that more-clustered communities were easily invaded [43], and the more divergent communities were less invaded by alien plants [53]. When we only considered the nearest phylogenetic relationship of native plants, we found that the more divergent the phylogenetic structure of native communities in AL, the higher the invasibility of habitat. The fact that phylogenetically dispersed native communities are more susceptible to invasion is usually the result of more intense competition between closely related species than between distant species [54], and competition may be more important for communities with phylogenetic structure dispersion than environmental filtering. The farther the phylogenetic distance between native communities, the lower the degree of ecological niche overlap and the weaker the competition. Our results are consistent with the study on *Serratia marcescens* invasion communities by Ketola [55] but are inconsistent with the study on *Ageratina adenophora* (Sprengel) R. M. King & H. Robinson [56]. SES.MPD and SES.MNTD are indices to measure the structure of a community. On the one hand, the opposite direction of these two indices can be effectively explained from the hypothetical model of adding species to a simple phylogenetic tree [57]. When a phylogenetic tree has multiple branching points, its values will be artificially increased or decreased [58]. The increase in species richness will generally lead to an increase in mean pairwise distance. On the other hand, phylogenetic distance is a proxy for resource-/niche-use complementarity and is sensitive to the phylogenetic history of communities, and this effect may blur the relationship between competition and phylogenetic correlation, which may also be one of the reasons why evidence of the role of phylogenetic distance in invasion is mixed [59].

The invasibility of EP was significantly positively correlated to the MNTD between invasive alien plants and native plants; our findings are consistent with those of Strauss [47] and provide supporting evidence for Darwin’s naturalization hypothesis. The greater the phylogenetic distance between invasive alien plants and native plants in EP, the higher the invasibility of the habitat. First, Darwin’s naturalization hypothesis proposed that species that are more closely related are more ecologically similar and more competitive, so alien species that are distantly related to native species may be more likely to invade successfully. Unlike AL, plants under EP contain more ferns. While ferns were removed in this analysis, the understory native vegetation was relatively simple, and the invasive alien plants related to native plants may be excluded due to restriction of similarity [59], so alien plants that are not similar to native plants are more likely to invade successfully.

Disturbance factors including the average human flow and average traffic flow significantly affected the invasibility of all three habitats, but the direction of impacts was different. Average traffic flow was positively correlated in EP and NF. This can be explained by the disturbance hypothesis—more frequent human interference causes the habitat to produce ecological niches suitable for the growth of alien plants and thus promotes invasion. On the other hand, people and vehicles are also carriers of invasive alien plant propagules and therefore act as promoters of invasion [60,61]. Moreover, we also found the average human flow was negatively correlated with the invasibility of all three habitats. According to the disturbance hypothesis, the more frequent the human activity, the more vulnerable habitats are to invasion [62]. The intermediate disturbance hypothesis supposes moderate levels of disturbance (either frequency or intensity) are expected to enhance species richness [63]. The competitive exclusion between species under low disturbance results in low diversity, and only pioneer species survive at high disturbance. When disturbance reaches a moderate level, primary and pioneer species can coexist, thus achieving higher biodiversity [64]. Our research found that *Chromolaena odorata* and *Bidens pilosa* often formed dominant communities in abandoned lands. *Chromolaena odorata* also formed dominant communities in the eucalyptus plantation in Encheng National Nature Reserve. It is noteworthy that disturbance in this study is represented by the average human flow and traffic flow, which are all indirect indexes. In the future, the direct disturbance index should be considered in control experiments. Our results still can reflect the impact of disturbance on invasion.

## 5. Conclusions

Our study found that the invasibility in the three habitats differed among habitat types—phylogenetic factors affected the invasibility of AL and EP, disturbance factors affected the invasibility of the three habitats, while native-plant diversity in the invaded habitats had a weak impact. Results of this study indicate that increasing the phylogenetic diversity of native plants can increase the resistance of the community in AL; otherwise, closer phylogenetic native plants should be selected to increase the competition between native plants and invasive plants in EP. Considering the limitations of our study, the invasibility index and disturbance factors such as the biomass proportion of invasive alien plants and the weed-clearing activity in EP should be considered in detail. This study enriches knowledge of the mechanisms of alien plant invasion into different habitats and provides a theoretical reference for habitat restoration in nature reserves. Results of this study may help to guide the management of invasive alien plants in the Encheng National Nature Reserve.

## Figures and Tables

**Table 1:** Habitat, locations, land-use, and functional zones of the nine research sites.

Habitat	Longitude	Latitude	Altitude	Functional Zone	Land-Use
EP	107.228	22.663	217	Experimental	The trees are 5 years old, planted at a density of 0.288 trees/m^2^, and the only other trees are *Ficus auriculata* Lour.
	106.979	22.793	368	Experimental	The trees are 7 years old, with a density of 0.188 trees/m^2^, and the other trees are mainly *Vernicia fordii* (Hemsl.) Airy Shaw and *Litsea monopetala* (Roxb.) Pers.
	106.978	22.798	378.53	Experimental	The trees are 8 years old, with a density of 0.342 trees/m^2^, and the understory shrubs are mainly *Mussaenda pubescens* W. T. Aiton.
AL	107.224	22.720	219.82	Experimental	Originally a reservoir, then planted with rice, abandoned for 2 years, no trees and shrubs, with grazing activities.
	107.094	22.778	229	Experimental	Originally planted with sugar cane crops, the abandonment time is unknown, without trees and shrubs, with grazing activities.
	107.088	22.756	132.63	Core	*Gardenia jasminoides* Ellis was planted before abandonment and abandoned for 3 years without trees and shrubs.
NF	107.060	22.789	267.7	Experimental	The trees are mainly *Litsea pungens* Hemsl. and *Maesa balansae* Mez.
	107.088	22.756	168.19	Core	Shrubs are mainly *Fordia cauliflora* Hemsl., and trees include *Micromelum integerrimum* (Buch.-Ham.) Roem.
	107.121	22.758	178.88	Experimental	The trees are mostly *Cipadessa baccifera* (Roth.) Miq, which were sparse.

EP, eucalyptus plantation; AL, abandoned land; and NF, natural secondary forest.

**Table 2:** List of invasive alien plants.

Family	Species	Life Form	Origin	Distribution
Asteraceae	*Chromolaena odorata* (L.) R. M. King & H. Robinson	Herbs, perennial	America	AL, EP, NF
	*Bidens Pilosa* L.	Herbs, annual	America	AL, EP, NF
	*Ageratum conyzoides* L.	Herbs, annual	Tropical America	AL, EP
	*Synedrella nodiflora* (L.) Gaertn.	Herbs, annual	South America	EP
	*Erigeron canadensis* L.	Herbs, annual	North America	AL
Fabaceae	*Mimosa bimucronata* (DC.) Kuntze	Shrubs, deciduous	Tropical America	EP
Oxalidaceae	*Oxalis corymbosa* DC.	Herbs, perennial	Tropical America	EP
Malvaceae	*Sida acuta* Burm. F.	Subshrubs or herbs erect	Tropical America	NF
Rubiaceae	*Spermacoce alata* Aublet	Herbs, perennial	Tropical America	EP
Poaceae	*Paspalum conjugatum* Berg.	Herbs, perennial	Tropical America	AL, EP
Solanaceae	*Solanum torvum* Swartz	Shrubs	Caribbean	NF
Euphorbiaceae	*Euphorbia hypericifolia* L.	Herbs, annual	America	NF

**Table 3:** Stepwise regression models of habitat invasibility.

Habitat	Stepwise Regression Equation	R^2^	*p*	MSE	AIC
AL	RA=1.379−0.112×SES.MPD+0.154×SES.MNTD−0.057×average traffic flow−0.42×average human flow	0.852	<0.001	0.019	−132.522
EP	RA=1.185+1.022×MNTD+0.191×average traffic flow−1.228× average human flow-	0.588	<0.001	0.038	−109.739
NF	RA=0.092+1.169×average traffic flow−5.099× average human flow	0.339	<0.001	0.011	−154.84

EP, eucalyptus plantation; AL, abandoned land; NF, natural secondary forest; RA, relative abundance of alien invasive plants in each habitat; SES.MNTD, standardized effect sizes of mean nearest taxon distance; SES.MPD, standardized effect sizes of mean pairwise distance; and MNTD, mean nearest taxon distance between invasive alien plants and native plants.

## Data Availability

The data presented in this study are available on request from the corresponding author (e-mail: zhaocy@craes.org.cn).
